# Orbitofrontal Reality Filtering

**DOI:** 10.3389/fnbeh.2013.00067

**Published:** 2013-06-10

**Authors:** Armin Schnider

**Affiliations:** ^1^Division of Neurorehabilitation, Department of Clinical Neurosciences, University Hospital, University of Geneva, Geneva, Switzerland

**Keywords:** orbitofrontal cortex, confabulations, reward system, reality monitoring, continuous recognition

## Abstract

Decades of research have deepened our understanding of how the brain forms memories and uses them to build our mental past and future. But how does it determine whether an evoked memory refers to the present and can be acted upon? The study of patients who confuse reality, as evident from confabulation and disorientation, has opened ways to explore this vital capacity. Results indicate that the brain recurs to a phylogenetically old faculty of the orbitofrontal cortex – extinction – and structures of the reward system to keep thought and behavior in phase with reality.

## Introduction

Since the early studies of patients who lost the ability to acquire new memories after damage to the hippocampal area (Scoville and Milner, 1957), the understanding of brain processes allowing the storage of information has been immensely refined (Squire and Wixted, 2011). Memories eventually become independent of the hippocampus, are stored and processed in distributed cortical areas (Squire and Wixted, 2011). Forging plans for the future appears to involve very much the same neural structures that are necessary to store information; building a mental future is very similar to constructing a personal past (Schacter et al., 2007). This raises the question of this review: how does the brain determine whether an upcoming thought pertains to “now”? How do we sense what our current duties are, what day it is, and what ideas we may currently act upon?

## Lost Sense of Reality

Brain damage may deprive humans of the ability to sense where they are and what their role is, while leaving other mental capacities intact. Hospitalized patients insisting on their obligation to organize a funeral or to resume military duties were already documented a century ago (Korsakoff, 1891; Kalberlah, 1904). A patient of ours, a retired psychiatrist hospitalized after rupture of aneurysm of the anterior communicating artery, was convinced that she was actually working as a psychiatrist at our clinic and repeatedly left therapy sessions in the conviction that she had to see patients (Schnider et al., 2005a; Schnider, 2008). A young lawyer, suffering from limbic encephalitis, desperately searched for her files, convinced that she was expected at court (Nahum et al., 2010). Both insisted on their resented reality although the hospital environment and therapy sessions should have indicated to them that they were not at work and that their ideas were wrong.

These patients had typical behaviorally spontaneous confabulation (Schnider, 2008), a syndrome first described by Korsakoff (1891) more than 100 years ago: the patients act according to false ideas that can mostly be traced back to real experiences (mostly habits), justify their actions with apparently invented stories (confabulations), are amnesic, and are disoriented regarding time, place, and their current situation.

**Figure 1:**
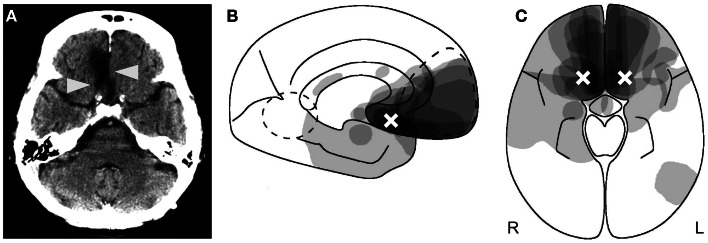
**Anatomy of reality filtering**. **(A)** Typical orbitofrontal lesion causing reality confusion. In this case, the right gyrus rectus is destroyed (arrowheads) following rupture of an aneurysm of the anterior communicating artery. **(B,C)** Superimposition of the lesions of 14 patients who confused reality for weeks to months. **(B)** Sagittal cut; **(C)** axial cut. As indicated by the shades of gray, maximal lesion overlap was in the posterior medial orbitofrontal area. The white crosses indicate the area of peak activity in healthy subjects who performed a similar task as the one on which the reality-confusing patients failed. **(B,C)** Reproduced from Schnider (2008), with permission.

The disorder was initially described in alcoholic, malnourished people suffering from the Wernicke–Korsakoff syndrome and in subjects having traumatic brain injury (Korsakoff, 1891; Bonhoeffer, 1901; Kalberlah, 1904). Nowadays, it is most frequently reported after rupture of an aneurysm of the anterior communicating artery, traumatic brain injury, or encephalitis (Schnider, 2008). The critical variable is not the type of brain damage, but the location: all hitherto described patients with circumscribed lesions – apart from dementia or a confusional state – had damage to the posterior medial orbitofrontal cortex or of brain regions directly connected with it (Figure 1) (Schnider et al., 1996b; Schnider and Ptak, 1999; Gilboa and Moscovitch, 2002; Schnider, 2008). Posterior lesion extension determines how much information the patients can store but is irrelevant for reality confusion as described above (Schnider, 2008). Hippocampal damage is often absent but may also be maximal (Schnider and Ptak, 1999; Nahum et al., 2010). Extremely severe amnesia and extended hippocampal damage do not protect against confabulation, as recently claimed (Dalla Barba and La Corte, 2013).

## Limbic Control of Memory and Reality

These clinical observations alone reveal a fundamental organizing principle of the limbic system’s contribution to memory control: while the posterior limbic system with the hippocampus is necessary for long-term encoding of memories (Squire et al., 2004), possibly also the retrieval of episodic details (Moscovitch et al., 2005), the anterior limbic system with the posterior medial orbitofrontal cortex is critical for the sense of whether an activated memory relates to the “now” or not. It signals when an activated memory does not pertain to ongoing reality (Schnider, 2008). It thus prevents behavior from being based on fantasies, that is, ideas that do not refer to the present.

## Sense of Time

Reality-confusing patients also have a disturbed sense for the “now,” the “perceived or psychological present,” defined as the duration of an experiential process, suggested to take about 0.1–5 s (Fraisse, 1984). In comparison to non-confabulating amnesics and healthy controls, reality-confusing patients failed to discriminate short, temporally overlapping intervals in the range of 0.2–3 s (Schnider, 2000). Similar findings have been obtained in patients with damage of the basal ganglia or cerebellum (Ivry and Keele, 1989; Gibbon et al., 1997; Riesen and Schnider, 2001). These structures were also activated, together with dorsolateral prefrontal and parietal cortex, in functional magnetic resonance imaging (fMRI) of time estimation and reproduction (Bueti et al., 2008). Damage to these structures does not induce reality confusion. It might, therefore, be that the disturbed discrimination within the psychological present in reality-confusing patients either reflects an independent time function, not causally related to reality confusion, or that the posterior medial orbitofrontal cortex, which is difficult to discern in fMRI due to artifacts, holds a central role in the sense of the “now,” but requires participation of association areas. The precise significance of the finding is as yet unclear.

## Exploring the Sense of Reality

**Figure 2:**
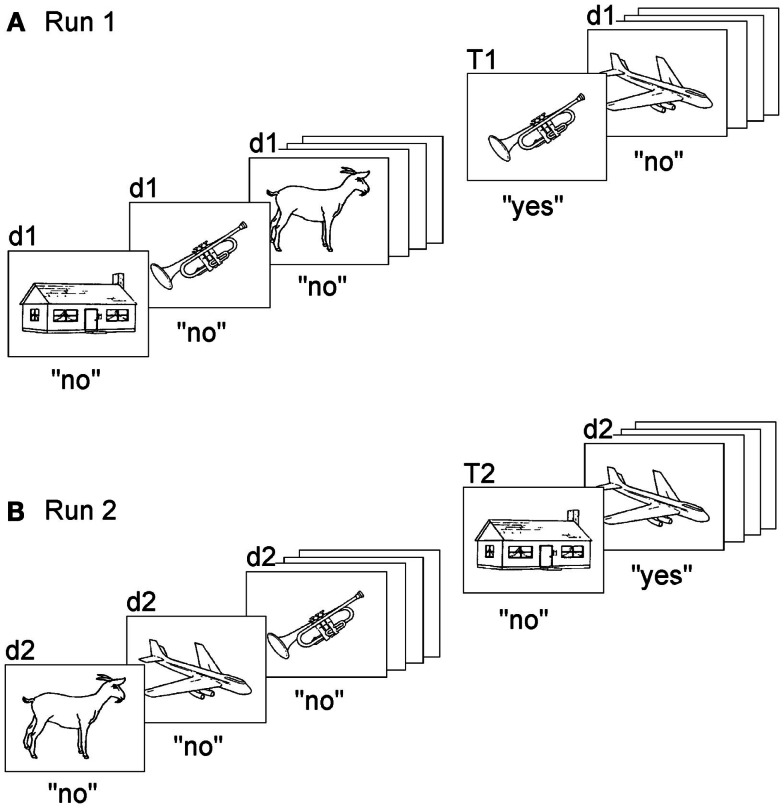
**Task to measure the sense of present reality**. Subjects make two runs (or more) of a continuous recognition task, each run composed of the same set of pictures. Subjects have to indicate, in both runs, only repetitions within the ongoing run (original version: Schnider et al., 1996a). **(A)** The first run demands learning and recognition and can be solved on the basis of familiarity alone. **(B)** In the second run, all items are already familiar. The task now demands the ability to distinguish between memories that pertain to the ongoing run (repetitions within the run, T2) and memories that do not (d2; not previously presented within the run, albeit familiar from the first run). Confabulating patients had a steep increase of false positives in response to d2 stimuli. “d” denotes “distracters,” i.e., pictures’ first appearance within a run; “T” denotes targets, i.e., repeated pictures within the run. “d1” and “T1” are stimuli presented in the first run, “d2” and “T2” are stimuli of the second run. “Yes” and “no” indicate correct responses. Illustration reproduced from Schnider (2008), with permission.

In any case, the temporal difficulty of reality-confusing patients transcends the perception of the present moment: they apparently fail to place themselves correctly in time and space. They act as obstinately on ideas that have no relation with the present as on ideas that refer to the present. How might one experimentally seize this intrusion of thoughts, which have no relation with the present, into their concept of reality and actions? We were lucky to develop a task, which tests the sense for memories’ relation with the “now” and which has proved very reliable in separating reality-confusing patients from other amnesic subjects (Schnider et al., 1996a; Schnider and Ptak, 1999; Nahum et al., 2012). The task uses repeated runs of a continuous recognition test, in which subjects see a long series of pictures and have to indicate picture recurrences within the ongoing run (Figure 2). When subjects do such a task for the first time, they can recognize picture repetitions on the sole basis of familiarity (Figure 2A). Healthy subjects performing such a first run activated the hippocampal area (Schnider et al., 2000b).

As subjects repeat the task, always composed of the same picture series, familiarity alone is not sufficient anymore; all items look familiar. Thus, the recognition of a repetition within the ongoing run now requires the ability to sense whether a picture was previously seen within the ongoing run (the “present reality” of the ongoing run) or a previous run; it requires reality filtering (Figure 2B). Despite this requirement, healthy subjects perform the task intuitively, with no particular effort: reaction times are similar to the first run and errors (false positive responses) are very scarce (Schnider et al., 2002; Wahlen et al., 2011). Indeed, it has proved very difficult to develop a task version which lowered performance of healthy subjects (Schnider et al., 2010).

When healthy subjects performed repeated runs of this task, they had activation of the posterior medial orbitofrontal cortex, area 13 (Figures 1B,C) (Schnider et al., 2000b), which corresponds to the area of maximal damage in patients who confuse reality.

The task structurally resembles well-known source memory tasks requiring attribution of stimuli to previous task stages, such as, the exclusion condition of the process dissociation procedure (Jacoby, 1991), in that it has multiple runs. Despite this resemblance, the processes involved in such tasks are very different from ours: they require conscious, effortful monitoring (Jacoby, 1991), activate the dorsolateral prefrontal (rather than orbitofrontal) cortex (Rugg et al., 2003), and have no predictive value for the occurrence of behaviorally spontaneous confabulation (Johnson et al., 1997). Reality filtering is not about knowing to what episode in the past a memory refers but whether it pertains to present reality or not.

Our reality-filtering task, as easy as it may be for healthy subjects, proved an insurmountable challenge for reality-confusing patients, even at much longer intervals between the runs (30–60 min) than in healthy subjects (1 min). While healthy subjects and non-confabulating amnesics maintained performance over repeated runs, reality-confusing patients had a sharp increase of false positive responses: they believed increasingly more often that they had already seen pictures within the ongoing run, which in reality appeared for the first time within the run (Schnider et al., 1996a; Schnider and Ptak, 1999; Nahum et al., 2012). This increase of false positives was also tightly associated with the degree of disorientation, that is, the number of false answers to questions about current time, place, or situation (Schnider et al., 1996b; Nahum et al., 2012). Recovery of the ability to sense that familiar items had not yet appeared within the ongoing run was individually predictive of the recovery of the sense of reality with cessation of confabulations and inappropriate acts and re-installment of correct orientation (Schnider et al., 2000a). The increase of false positives in reality-confusing patients indicates that orbitofrontal reality-filtering functions by exclusion: it signals when a memory does not relate to current reality.

## Preconscious Reality Filtering

The conviction that healthy subjects, but also reality-confusing patients hold in their concept of current reality – the present day, their current role and location, etc. – suggests that reality filtering is an early process, which precedes conscious control. Experimental evidence supports this notion. When healthy subjects performed a similar task while their brain activity was observed with electroencephalography, processing of new and repeated items in the first run, which requires learning and recognition, differed over posterior electrodes at around 400–600 ms (Schnider et al., 2002; Wahlen et al., 2011). By contrast, processing of new items in the second run, which requires reality filtering, induced a strikingly different electrocortical potential than all other stimuli of the first and second run: at 200–300 ms, they did not evoke a negative frontal potential common to all other stimuli. Thus, correct processing of the stimuli on which reality-confusing patients had failed (first presentations within the second run) differed from all other stimuli at an early stage, before processes of recognition set in. In other words, even before we recognize the precise content of an upcoming memory (thought), the orbitofrontal cortex has already decided whether it refers to ongoing reality or not.

Spatio-temporal analysis of the electrical activity over the whole brain indicated that the absence of the negative potential reflected the fact that new stimuli of the second run skipped a processing stage common to all other stimuli, which was characterized by a particularly extended neocortical, temporo-parietal area of synchronous activity (Schnider et al., 2002; Schnider, 2003; Wahlen et al., 2011), as indicated by source estimation (Michel et al., 2004). This suggests that, whenever a memory is activated that does not relate to reality (fantasy), neocortical synchronization is transiently inhibited at 200–300 ms (Schnider, 2003). Fantasies would thus assume a different electrocortical format than thoughts that pertain to ongoing reality and that have passed through the stage of extended neocortical activation (Schnider, 2008).

**Figure 3:**
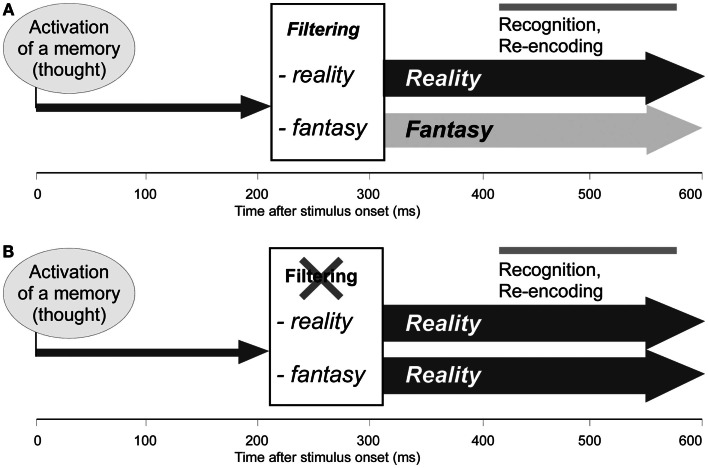
**Functional model of the orbitofrontal reality filter**. **(A)** Normal function: 200–300 ms after activation of a memory (thought), reality filtering sets in, inhibiting extended neocortical activation when the upcoming memory does not relate to ongoing reality. At 400–600 ms, the activated memory (thought) is recognized and again encoded (Schnider et al., 2002; Wahlen et al., 2011). **(B)** Hypothetical dysfunction of reality filtering in reality-confusing patients: memories (thoughts) are normally activated, but are not checked regarding their relation with reality; all memories are activated, and later recognized and re-encoded, as if they related to ongoing reality. Adapted from Schnider (2008), with permission.

This sequence of processes – first reality filtering, then recognition and re-encoding of memories (thoughts), as depicted in Figure 3A – not only ensures that we distinguish between memories that pertain to ongoing reality and memories that do not, but also that we know tomorrow whether we have really experienced a situation today or only thought about it; as these thoughts (memories) are re-encoded, they are labeled as referring to reality or as a fantasy (Schnider, 2008). In reality-confusing patients (Figure 3B), memories that do not relate to reality are not filtered and thus assume the same format as memories relating to reality. Any upcoming thought, be it a memory of the past, a thought about the present, or a plan for the future, is sensed as if it referred to the present. Depending on the evoked memories, patients’ behavior sometimes agrees, sometimes disagrees with reality. As far as the patients encode their own thoughts, they experience them as if they referred to reality and may, therefore, subsequently produce false statements about the past, present, or future. In agreement with this interpretation, confabulating patients may recall events that they have simply talked about, as if they had really experienced them (Schnider et al., 2005a).

Reality filtering is not limited to visual information: we observed similar orbitofrontal activation in functional imaging with visually presented verbal or non-verbal visual material (Treyer et al., 2003) as well as with auditorily presented words (Treyer et al., 2006). The process is precise: the electrocortical signal was much more distinct when stimuli between the runs were identical with, rather than only resembled previously presented ones (Wahlen et al., 2011). Thus, reality filtering seems to be challenged particularly when present reality is very similar to a past reality. Finally, the process is distinct from other memory control mechanisms: to recognize that a stimulus only resembles, but is not identical with a previously seen stimulus (task described by Gilboa et al., 2006) also evokes an electrocortical signal at 200–300 ms but with inverse polarity than the one produced by reality filtering (Wahlen et al., 2011). That is, the recognition of a memory’s concordance with the present (reality filtering) dissociates from the recognition of its concordance with the past (its content).

## Orbitofrontal Cortex and Reality Filtering

The posterior medial orbitofrontal cortex is a phylogenetically old and ontogenetically consistent structure (Chiavaras et al., 2001). One may, therefore, wonder what specific faculty enables it to assume the role of a reality filter. Reality-confusing patients fail to adapt to the fact that their anticipations never come true: the hospitalized psychiatrist did not find her expected patients, the lawyer did not find the colleagues and judges she expected to meet. Yet, both continued to act according to such anticipations – based on habits – as if they were still valid. This behavior is reminiscent of animals continuing to choose a conditioned stimulus, which was previously followed by reward, even after this association has proved to be no longer valid. The ability to learn from the fact that a stimulus is no longer followed by reward and to abandon a previously valid stimulus-outcome association is called extinction (Pavlov, 1927; Ouyang and Thomas, 2005). In non-human primates, lesions of the posterior medial orbitofrontal cortex, corresponding to area 13, induced a specific deficit of extinction, unlike damage to any other region of the prefrontal lobes (Butter, 1969). Single cell recordings showed that this area contains a particularly high density of neurons that specifically discharge when an expected reward fails to be delivered (Rosenkilde et al., 1981). Our hypothesis is that the brain uses this neural signal to label an upcoming memory as not pertaining to ongoing reality, that is, as a fantasy. The neurons producing this signal might be appropriately described as “reality neurons.”

Clinical evidence supports this hypothesis: we asked a group of amnesic subjects to perform a reversal learning task, in which they had to predict which one of two faces would have a target stimulus on the nose (Nahum et al., 2009). Reality-confusing patients did not differ from other amnesics in their ability to learn the association, but they had significantly more difficulty in switching to the alternate face after trials indicating absence of the target stimulus. Over all patients, this difficulty, but not other cognitive measures, highly correlated with the degree of disorientation.

The data indicate that, rather than invoking high-level monitoring mechanisms, the brain uses a phylogenetically old capacity, already available to primitive creatures like aplysia (Hawkins et al., 2006) and drosophila (Schwaerzel et al., 2002), to keep thought and behavior in phase with reality: it uses the neural signal that also underlies behavioral extinction. So, evolution did not have to devise a separate mechanism to assure the behaviorally appropriate use of an ever-increasing stock of memories in higher species like humans.

## Reality Check and Reward System

These results point to a hitherto unappreciated role of the orbitofrontal cortex and the reward system in reality filtering beyond processing the pleasure associated with outcomes. Indeed, orbitofrontal activity does not depend on the prospect of pleasure: the human orbitofrontal cortex is also activated when anticipating and monitoring neutral events devoid of any tangible reward value (Schnider et al., 2005b). In such a task, the non-occurrence of anticipated outcomes induced a distinct electrocortical signal, which occurred in the same period (200–300 ms) and with a similar configuration (frontal positivity) as the signal produced in reality filtering (Schnider et al., 2007). Indeed, it appears that, in humans, posterior orbitofrontal activity in response to the non-delivery of expected reward is driven much more by the need to adapt behavior than the sole absence of the reward; when there was no need to adapt behavior, absence of reward did not activate this area (Nahum et al., 2011). While these results do not question the role of the orbitofrontal cortex in hedonic processing (Kringelbach, 2005), decision-making, and optimizing behavior (Bechara et al., 1997; Wallis, 2007), they underscore that the orbitofrontal cortex contains the neural apparatus allowing it to function as a generic reality-filtering system, irrespective of whether reward is at stake or not.

The similarity between reality filtering and reward processing extends to transmitter systems. While select orbitofrontal neurons increase firing when an anticipated reward fails to occur (Rosenkilde et al., 1981; Thorpe et al., 1983), dopaminergic neurons in the nigro-striatal system transiently decrease their firing (Schultz et al., 1997; Schultz, 2007). A “hyper-dopaminergic” state would, accordingly, be expected to impair reality filtering. This is what we found (Schnider et al., 2010): when healthy subjects performed a very difficult version of our reality-filtering task under the influence of l-DOPA, which is transformed to dopamine in the brain, they produced specifically more false positive responses than when they received a dopamine antagonist (risperidone). Thus, reality filtering, similar to reward processing, is under dopaminergic modulation. Of note, a hyper-dopaminergic state has also been suspected to underlie schizophrenia, another disorder characterized by reality confusion (Howes and Kapur, 2009). Actively hallucinating schizophrenic patients failed in our reality-filtering task (Badcock et al., 2005). Pervasive confabulations were described in such patients in the nineteenth century (Kraepelin, 1887/88) before the advent of neuroleptics (dopamine antagonists) in the late 1940s. The implication of diverse dopamine sub-systems and other transmitter systems in reward and outcome processing is a topic of current research (Schultz and Dickinson, 2000; Bromberg-Martin et al., 2010); their role for reality filtering is entirely unknown.

Available data suggest a link between orbitofrontal activity and subcortical dopaminergic transmission. The orbitofrontal cortex projects onto dopaminergic neurons in the midbrain (Joel and Weiner, 2000), which, in turn, project onto, and modulate activity in frontal-subcortical loops that connect specific areas of the prefrontal lobes, including the orbitofrontal cortex, with themselves and other frontal areas (Alexander et al., 1986). By transiently inhibiting dopaminergic neurons, the orbitofrontal cortex might thus modulate activity in subcortical connections and convey the signal inhibiting neocortical synchronization when an upcoming memory does not relate to reality. In agreement with this, we observed activation of a loop connecting the orbitofrontal cortex with the striatum, substantia nigra, and the medial thalamus when healthy subjects performed a more sensitive version of the reality-filtering task containing different stimulus types (Treyer et al., 2003). This result, together with the pharmacological evidence, suggests that reality filtering not only relies on the same orbitofrontal signal, but also on the same circuitry as the one used to signal that an anticipated reward failed to occur. We surmise that reality filtering is a specific instance of “reward” processing: it refers to all types of outcomes, irrespective of hedonic value, and specifically processes the situation that anticipated events fail to happen.

## Comparison with Other Hypotheses

Orbitofrontal reality filtering, as described in this review, does not yet have the status of a distinct, acknowledged brain function. Accordingly, there is no hypothesis to compare it with. By contrast, the two obvious disorders resulting from its dysfunction – disorientation and confabulation – have received the attention of cognitive models.

Disorientation has been linked to amnesia and perception: an uninterrupted flow of memories and correct perception of the environment would be necessary to maintain orientation in time and space (Kraepelin, 1909; Benton et al., 1964; High et al., 1990). Our data only partially support this notion: the severity of amnesia, as measured with a continuous recognition task, is only weakly associated with disorientation (Schnider et al., 1996b; Schnider, 2008). However, early authors also speculated that there might be a distinct function of orientation (Bleuler, 1923) and Jaspers (1973) separated “delusional disorientation” in patients with full consciousness as a distinct form. Deficient reality filtering might be the mechanism they thought of.

Confabulations have been the topic of diverse hypotheses. Most of them tried to explain confabulations as a verbal phenomenon, irrespective of inappropriate behavior or disorientation. The question at the center of these hypotheses was: “What makes patients tell incorrect stories and fabricate false responses to questions?” The question at the basis of our studies was: “why do the patients confuse reality?”

The first question refers to two forms of confabulation: (1) Intrusions in memory tests (simple provoked confabulations). These dissociate from all other forms of confabulation and are independent of reality confusion (Schnider et al., 1996a; Nahum et al., 2012). (2) Momentary confabulations (also called out-of-embarrassment confabulations) (Bonhoeffer, 1901; Van der Horst, 1932; Schnider, 2008) that patients produce in discussions or in response to questions. It is the most commonly reported form of confabulation. It need not be accompanied by disorientation. Only a relatively small proportion of patients who produce momentary confabulations have deficient reality filtering (Nahum et al., 2012).

The second question refers to two other forms: (3) behaviorally spontaneous confabulation, as defined above – the topic of this review. It can be conceived as a specific subform of momentary confabulations, namely, the form caused by reality confusion, as evident from inappropriate acts in accordance with the confabulations and disorientation. (4) Fantastic confabulations, which defy any sense of plausibility. These are rare and occur in severe confusion, dementia, or psychosis (Schnider, 2008).

Most hypotheses did not distinguish between forms of confabulations. Thus, they have differing significance for the explanation of behaviorally spontaneous confabulation and reality confusion. The most prominent hypotheses can be summarized as follows: (1) Confabulations emanate from a combination of amnesia with frontal executive failures. The hypothesis stems from the observation that executive functions may recover in parallel with the cessation of confabulations (Papagno and Baddeley, 1997; Nys et al., 2004) and that confabulations in cohorts of brain damaged patients are associated with executive dysfunction (Cunningham et al., 1997). However, patients with behaviorally spontaneous confabulation did not differ from similarly severe non-confabulating amnesics with regards to executive functions (Schnider et al., 1996a; Schnider and Ptak, 1999; Nahum et al., 2012). (2) Confabulations reflect a desire to fill gaps in memory or may compensate for the embarrassment of being unable to respond to questions (Flament, 1957; Conway and Tacci, 1996; Fotopoulou et al., 2008). This mechanism appears to account for a certain proportion of momentary confabulations, but was not associated with behaviorally spontaneous confabulation or disorientation (Schnider et al., 1996a; Nahum et al., 2012). Nonetheless, it may be that this mechanism explains the content of false ideas, possibly also the tendency to talk about them. If reality filtering fails, then patients not only talk about, but also act in accordance with these ideas. (3) Confabulations emanate from deficient monitoring of the source (spatial, temporal, personal context) of memories (Johnson and Raye, 1998). Insofar as this function has been defined by experimental procedures, which typically require effortful and conscious monitoring (see process dissociation procedure, above), it has failed to differentiate confabulating from non-confabulating patients (own unpublished data and Johnson et al., 1997). Conversely, reality filtering, which precedes the re-encoding of thoughts, is likely to be a prerequisite for later source monitoring. (4) Confabulations reflect insufficient monitoring of memories’ content (Moscovitch and Melo, 1997; Gilboa et al., 2006). Confabulating patients who failed both in our reality-filtering task and a task requiring fine distinction between closely similar items were indeed described (Gilboa et al., 2006). We found that these two challenges evoked different cortical processes (Wahlen et al., 2011). It may be that in select patient groups, both processes may contribute to the occurrence and the content of confabulations. (5) Confabulations results from a deficient temporal tag of memories or reflect temporally displaced consciousness (Van der Horst, 1932; Talland, 1961; Dalla Barba, 2002; Dalla Barba and La Corte, 2013). The hypothesis is based on the observation that confabulations very often are rooted in patients’ true experiences and falsely recombine elements of real events. While these authors never proposed a way to experimentally verify the hypothesis, the idea is entirely compatible with the concept of reality filtering, but with a twist: reality filtering only explains confabulations emanating from reality confusion and, therefore, only a certain proportion of confabulations.

## Limitations of the Task

The continuous recognition task used to test reality filtering, as predictive as it has been in clinical practice and as consistent imaging and electrophysiological results have been, has its limitations. First, it may fail to seize memory confusion in patients with extremely severe amnesia who fail to encode any information in the first run (Schnider et al., 1996a). Such patients still failed in the extinction task (Nahum et al., 2009; Schnider et al., 2013). Second, like any cognitive test, a subject’s strategy may influence results. One of our patients was so skeptical about the difficulties of the second run that he rejected all items as being repetitions. When the task was repeated indicating that not only his correct rejections, but also his correct recognitions would be counted, the typical pattern of memory confusion became apparent (Ptak and Schnider, 1999). Third, in our studies, patients were matched according to the severity of amnesia. There are indications that, if patients are selected according to a single etiology, irrespective of the severity of cognitive deficits, the task may not be very predictive of reality confusion (Joray et al., 2004; Gilboa et al., 2006). Fourth, the most important limitation of the task is its specificity. As this review should make clear, it has been developed to measure reality confusion; it is not a predictor of all forms of confabulation. This limitation, of course, applies to the whole concept of orbitofrontal reality filtering.

## Perspectives

A number of questions remain: what qualifies a real-world memory, composed of different modalities, as pertaining to reality? Is there a hierarchy of modalities, for example, with visual information, which is used in most experiments, prevailing over tactile information? Then, there are anatomical enigmas: first, virtually all patients confusing reality after an orbitofrontal lesion eventually regain the sense of reality, albeit sometimes only after many months (Schnider et al., 2000a, 2005a). This suggests that the neural apparatus ensuring reality filtering – presumably the outcome monitoring system – is redundantly organized or may undergo plastic changes after damage, similar to other systems. What brain areas and mechanism allow these patients to regain the sense of reality? Secondly, only a minority of people having a classic disease and orbitofrontal lesion actually suffer sustained reality confusion (Schnider, 2008); a typical lesion alone does not reliably predict the occurrence of reality confusion. Thus, do reality-confusing patients have the misfortune of concentrating their reality cells in the areas damaged by the common causes of behaviorally spontaneous confabulation? Is there a genetic predisposition for such an arrangement or for the ability to rapidly adapt thought to ongoing reality?

While some of these questions can be examined in humans, others need the precision of animal experimentation. The association between human reality filtering and the capacity to abandon previously valid anticipations suggests that extinction trials in reward tasks would be an appropriate animal model of human reality filtering. In contrast to extinction of fear memories (in which the animals gain access to a previously avoided stimulus) (LeDoux, 1996; Quirk et al., 2010) extinction of reward associations (in which the animals give up a previously rewarding association) has rarely been studied because such trials rapidly discourage the animals from participating. Experimentation would thus take longer. The investment may clearly be worth it: a better understanding of the processes underlying reality filtering might open new ways to treat diseases impairing the sense of reality.

## Conflict of Interest Statement

The author declares that the research was conducted in the absence of any commercial or financial relationships that could be construed as a potential conflict of interest.

## References

[B1] Alexander G. E. Delong M. R. Strick P. L. (1986). Parallel organization of functionally segregated circuits linking basal ganglia and cortex. Annu. Rev. Neurosci. 9, 357–381 10.1146/annurev.ne.09.030186.002041 3085570

[B2] Badcock J. C. Waters F. A. Maybery M. T. Michie P. T. (2005). Auditory hallucinations: failure to inhibit irrelevant memories. Cogn. Neuropsychiatry 10, 125–136 10.1080/13546800344000363 16571456

[B3] Bechara A. Damasio H. Tranel D. Damasio A. R. (1997). Deciding advantageously before knowing the advantageous strategy. Science 275, 1293–1295 10.1126/science.275.5304.1293 9036851

[B4] Benton A. L. Van Allen M. W. Fogel M. L. (1964). Temporal orientation in cerebral disease. J. Nerv. Ment. Dis. 139, 110–119 10.1097/00005053-196408000-00003 14206449

[B5] Bleuler E. (1923). Lehrbuch der Psychiatrie. Berlin: Julius Springer Verlag

[B6] Bonhoeffer K. (1901). Die Akuten Geisteskrankheiten des Gewohnheitstrinkers. Eine klinische Studie. Jena: Gustav Fischer

[B7] Bromberg-Martin E. S. Matsumoto M. Hikosaka O. (2010). Dopamine in motivational control: rewarding, aversive, and alerting. Neuron 68, 815–834 10.1016/j.neuron.2010.11.022 21144997 PMC3032992

[B8] Bueti D. Walsh V. Frith C. Rees G. (2008). Different brain circuits underlie motor and perceptual representations of temporal intervals. J. Cogn. Neurosci. 20, 204–214 10.1162/jocn.2008.20017 18275329

[B9] Butter C. M. (1969). Perseveration in extinction and in discrimination reversal tasks following selective frontal ablations in *Macaca mulatta*. Physiol. Behav. 4, 163–171 10.1016/0031-9384(69)90075-4

[B10] Chiavaras M. M. Legoualher G. Evans A. Petrides M. (2001). Three-dimensional probabilistic atlas of the human orbitofrontal sulci in standardized stereotaxic space. Neuroimage 13, 479–496 10.1006/nimg.2000.0641 11170813

[B11] Conway M. A. Tacci P. C. (1996). Motivated confabulation. Neurocase 2, 325–339 10.1093/neucas/2.4.325-a

[B12] Cunningham J. M. Pliskin N. H. Cassisi J. E. Tsang B. Rao S. M. (1997). Relationship between confabulation and measures of memory and executive function. J. Clin. Exp. Neuropsychol. 19, 867–877 10.1080/01688639708403767 9524881

[B13] Dalla Barba G. La Corte V. (2013). The hippocampus, a time machine that makes errors. Trends Cogn. Sci. (Regul. Ed.) 17, 102–104 10.1016/j.tics.2013.01.005 23415077

[B14] Dalla Barba G. F. (2002). Memory, Consciousness and Temporality. Boston: Kluwer Academic Publishers

[B15] Flament J. (1957). La fabulation dans le syndrome de Korsakov d’étiologie traumatique. Considérations cliniques, psycho-pathologiques et neuro-pathologiques à propos d’une observation de fabulation à caractère mythopathique. Acta Neurol. Belg. 57, 119–161 13424196

[B16] Fotopoulou A. Conway M. A. Tyrer S. Birchall D. Griffiths P. Solms M. (2008). Is the content of confabulation positive? An experimental study. Cortex 44, 764–772 10.1016/j.cortex.2007.03.001 18489957

[B17] Fraisse P. (1984). Perception and estimation of time. Annu. Rev. Psychol. 35, 1–36 10.1146/annurev.ps.35.020184.000245 6367623

[B18] Gibbon J. Malapani C. Dale C. L. Gallistel C. (1997). Toward a neurobiology of temporal cognition: advances and challenges. Curr. Opin. Neurobiol. 7, 170–184 10.1016/S0959-4388(97)80005-0 9142762

[B19] Gilboa A. Alain C. Stuss D. T. Melo B. Miller S. Moscovitch M. (2006). Mechanisms of spontaneous confabulations: a strategic retrieval account. Brain 129, 1399–1414 10.1093/brain/awl093 16638795

[B20] Gilboa A. Moscovitch M. (2002). “The cognitive neuroscience of confabulation: a review and a model,” in The Handbook of Memory Disorders, 2nd Edn, eds Baddeley A. Kopelman M. D. Wilson B. (West Sussex: John Wiley & Sons), 315–342

[B21] Hawkins R. D. Clark G. A. Kandel E. R. (2006). Operant conditioning of gill withdrawal in Aplysia. J. Neurosci. 26, 2443–2448 10.1523/JNEUROSCI.3294-05.2006 16510722 PMC6793659

[B22] High W. M. Levin H. S. Gary H. E. (1990). Recovery of orientation following closed head injury. J. Clin. Exp. Neuropsychol. 12, 703–714 10.1080/01688639008401013 2258432

[B23] Howes O. D. Kapur S. (2009). The dopamine hypothesis of schizophrenia: version III – the final common pathway. Schizophr. Bull. 35, 549–562 10.1093/schbul/sbp006 19325164 PMC2669582

[B24] Ivry R. B. Keele S. W. (1989). Timing functions of the cerebellum. J. Cogn. Neurosci. 1, 136–152 10.1162/jocn.1989.1.2.136 23968462

[B25] Jacoby L. L. (1991). A process dissociation framework: separating automatic from intentional uses of memory. J. Mem. Lang. 30, 513–541 10.1016/0749-596X(91)90025-F

[B26] Jaspers K. (1973). Allgemeine Psychopathologie. Berlin: Springer-Verlag

[B27] Joel D. Weiner I. (2000). The connections of the dopaminergic system with the striatum in rats and primates: an analysis with respect to the functional and compartmental organization of the striatum. Neuroscience 96, 451–474 10.1016/S0306-4522(99)00575-8 10717427

[B28] Johnson M. K. O’Connor M. Cantor J. (1997). Confabulation, memory deficits, and frontal dysfunction. Brain Cogn. 34, 189–206 10.1006/brcg.1997.0873 9220085

[B29] Johnson M. K. Raye C. L. (1998). False memories and confabulation. Trends Cogn. Sci. (Regul. Ed.) 2, 137–145 10.1016/S1364-6613(98)01152-8 21227110

[B30] Joray S. Herrmann F. Mulligan R. Schnider A. (2004). Mechanism of disorientation in Alzheimer’s disease. Eur. Neurol. 52, 193–197 10.1159/000082034 15539771

[B31] Kalberlah F. (1904). Ueber die acute Commotionspsychose, zugleich ein Beitrag zur Aetiologie des Korsakow’schen Symptomenkomplexes. Arch. Psychiatr. Nervenkr. 38, 402–438 10.1007/BF02173472

[B32] Korsakoff S. S. (1891). Erinnerungstäuschungen (Pseudoreminiscenzen) bei polyneuritischer Psychose. Allg. Z. Psychiatr. Psych. Med. 47, 390–410

[B33] Kraepelin E. (1909). Psychiatrie. Ein Lehrbuch für Studierende und Ärzte. I. Band, Allgemeine Psychiatrie. Leipzig: Johann Ambrosius Barth Verlag

[B34] Kraepelin E. (1887/88). Ueber Erinnerungsfälschungen. Arch. Psychiatr. Nervenkr. 17, 830–843 10.1007/BF02207467

[B35] Kringelbach M. L. (2005). The human orbitofrontal cortex: linking reward to hedonic experience. Nat. Rev. Neurosci. 6, 691–702 10.1038/nrn1747 16136173

[B36] LeDoux J. (1996). The Emotional Brain. The Mysterious Underpinnings of Emotional Life. New York: Simon & Schuster

[B37] Michel C. M. Murray M. M. Lantz G. Gonzalez S. Spinelli L. Grave De Peralta R. (2004). EEG source imaging. Clin. Neurophysiol. 115, 2195–2222 10.1016/j.clinph.2004.06.001 15351361

[B38] Moscovitch M. Melo B. (1997). Strategic retrieval and the frontal lobes: evidence from confabulation and amnesia. Neuropsychologia 35, 1017–1034 10.1016/S0028-3932(97)00028-6 9226662

[B39] Moscovitch M. Rosenbaum R. S. Gilboa A. Addis D. R. Westmacott R. Grady C. (2005). Functional neuroanatomy of remote episodic, semantic and spatial memory: a unified account based on multiple trace theory. J. Anat. 207, 35–66 10.1111/j.1469-7580.2005.00421.x 16011544 PMC1571502

[B40] Nahum L. Bouzerda-Wahlen A. Guggisberg A. Ptak R. Schnider A. (2012). Forms of confabulation: dissociations and associations. Neuropsychologia 50, 2524–2534 10.1016/j.neuropsychologia.2012.06.026 22781813

[B41] Nahum L. Gabriel D. Schnider A. (2011). Human processing of behaviorally relevant and irrelevant absence of expected rewards: a high-resolution ERP study. PLoS ONE 6:e16173 10.1371/journal.pone.0016173 21298049 PMC3029290

[B42] Nahum L. Ptak R. Leemann B. Lalive P. Schnider A. (2010). Behaviorally spontaneous confabulation in limbic encephalitis: the roles of strategic monitoring and reality filtering. J. Int. Neuropsychol. Soc. 16, 995–1005 10.1017/S1355617710000780 20719042

[B43] Nahum L. Ptak R. Leemann B. Schnider A. (2009). Disorientation, confabulation, and extinction capacity. Clues on how the brain creates reality. Biol. Psychiatry 65, 966–972 10.1016/j.biopsych.2009.01.007 19217613

[B44] Nys G. M. Van Zandvoort M. J. Roks G. Kappelle L. J. De Kort P. L. De Haan E. H. (2004). The role of executive functioning in spontaneous confabulation. Cogn. Behav. Neurol. 17, 213–218 15622017

[B45] Ouyang M. Thomas S. A. (2005). A requirement for memory retrieval during and after long-term extinction learning. Proc. Natl. Acad. Sci. U.S.A. 102, 9347–9352 10.1073/pnas.0502315102 15947076 PMC1166608

[B46] Papagno C. Baddeley A. (1997). Confabulation in a dysexecutive patient: implication for models of retrieval. Cortex 33, 743–752 10.1016/S0010-9452(08)70731-7 9444475

[B47] Pavlov P. I. (1927). Conditioned Reflexes: An Investigation of the Physiological Activity of the Cerebral Cortex, trans. Anrep G. V. (London: Oxford University Press) 10.5214/ans.0972-7531.1017309 PMC4116985 25205891

[B48] Ptak R. Schnider A. (1999). Spontaneous confabulations after orbitofrontal damage: the role of temporal context confusion and self-monitoring. Neurocase 5, 243–250 10.1080/13554799908402729

[B49] Quirk G. J. Pare D. Richardson R. Herry C. Monfils M. H. Schiller D. (2010). Erasing fear memories with extinction training. J. Neurosci. 30, 14993–14997 10.1523/JNEUROSCI.4268-10.2010 21068303 PMC3380534

[B50] Riesen J. Schnider A. (2001). Time estimation in Parkinson’s disease: normal long term estimation despite impaired short duration discrimination. J. Neurol. 248, 27–35 10.1007/s004150170266 11266017

[B51] Rosenkilde C. E. Bauer R. H. Fuster J. M. (1981). Single cell activity in ventral prefrontal cortex of behaving monkeys. Brain Res. 209, 375–394 10.1016/0006-8993(81)90160-8 7225799

[B52] Rugg M. D. Henson R. N. Robb W. G. (2003). Neural correlates of retrieval processing in the prefrontal cortex during recognition and exclusion tasks. Neuropsychologia 41, 40–52 10.1016/S0028-3932(02)00129-X 12427564

[B53] Schacter D. L. Addis D. R. Buckner R. L. (2007). Remembering the past to imagine the future: the prospective brain. Nat. Rev. Neurosci. 8, 657–661 10.1038/nrn2213 17700624

[B54] Schnider A. (2000). Spontaneous confabulations, disorientation, and the processing of ‘now’. Neuropsychologia 38, 175–185 10.1016/S0028-3932(99)00064-0 10660228

[B55] Schnider A. (2003). Spontaneous confabulation and the adaptation of thought to ongoing reality. Nat. Rev. Neurosci. 4, 662–671 10.1038/nrn1179 12894241

[B56] Schnider A. (2008). The Confabulating Mind. How the Brain Creates Reality. Oxford: Oxford University Press

[B57] Schnider A. Bonvallat J. Emond H. Leemann B. (2005a). Reality confusion in spontaneous confabulation. Neurology 65, 1117–1119 10.1212/01.wnl.0000178900.37611.8d 16217071

[B58] Schnider A. Treyer V. Buck A. (2005b). The human orbitofrontal cortex monitors outcomes even when no reward is at stake. Neuropsychologia 43, 316–323 10.1016/j.neuropsychologia.2004.07.003 15707609

[B59] Schnider A. Guggisberg A. Nahum L. Gabriel D. Morand S. (2010). Dopaminergic modulation of rapid reality adaptation in thinking. Neuroscience 167, 583–587 10.1016/j.neuroscience.2010.02.044 20219638

[B60] Schnider A. Mohr C. Morand S. Michel C. M. (2007). Early cortical response to behaviorally relevant absence of anticipated outcomes: a human event-related potential study. Neuroimage 35, 1348–1355 10.1016/j.neuroimage.2007.01.047 17355909

[B61] Schnider A. Nahum L. Pignat J. Leemann B. Lövblad K. Wissmeyer M. (2013). Isolated prospective confabulation in Wernicke-Korsakoff syndrome: a case for reality filtering. Neurocase 19, 90–104 10.1080/13554794.2011.654221 22512690

[B62] Schnider A. Ptak R. (1999). Spontaneous confabulators fail to suppress currently irrelevant memory traces. Nat. Neurosci. 2, 677–681 10.1038/10236 10404203

[B63] Schnider A. Ptak R. Von Däniken C. Remonda L. (2000a). Recovery from spontaneous confabulations parallels recovery of temporal confusion in memory. Neurology 55, 74–83 10.1212/WNL.55.1.74 10891909

[B64] Schnider A. Treyer V. Buck A. (2000b). Selection of currently relevant memories by the human posterior medial orbitofrontal cortex. J. Neurosci. 20, 5880–5884 10908632 10.1523/JNEUROSCI.20-15-05880.2000 PMC6772539

[B65] Schnider A. Valenza N. Morand S. Michel C. M. (2002). Early cortical distinction between memories that pertain to ongoing reality and memories that don’t. Cereb. Cortex 12, 54–61 10.1093/cercor/12.1.54 11734532

[B66] Schnider A. Von Däniken C. Gutbrod K. (1996a). The mechanisms of spontaneous and provoked confabulations. Brain 119, 1365–1375 10.1093/brain/119.4.1365 8813298

[B67] Schnider A. Von Däniken C. Gutbrod K. (1996b). Disorientation in amnesia. A confusion of memory traces. Brain 119, 1627–1632 10.1093/brain/119.5.1627 8931585

[B68] Schultz W. (2007). Multiple dopamine functions at different time courses. Annu. Rev. Neurosci. 30, 259–288 10.1146/annurev.neuro.28.061604.135722 17600522

[B69] Schultz W. Dayan P. Montague P. R. (1997). A neural substrate of prediction and reward. Science 275, 1593–1599 10.1126/science.275.5306.1593 9054347

[B70] Schultz W. Dickinson A. (2000). Neuronal coding of prediction errors. Annu. Rev. Neurosci. 23, 473–500 10.1146/annurev.neuro.23.1.473 10845072

[B71] Schwaerzel M. Heisenberg M. Zars T. (2002). Extinction antagonizes olfactory memory at the subcellular level. Neuron 35, 951–960 10.1016/S0896-6273(02)00832-2 12372288

[B72] Scoville W. B. Milner B. (1957). Loss of recent memory after bilateral hippocampal lesions. J. Neurol. Neurosurg. Psychiatr. 20, 11–21 10.1136/jnnp.20.1.11 13406589 PMC497229

[B73] Squire L. R. Stark C. E. Clark R. E. (2004). The medial temporal lobe. Annu. Rev. Neurosci. 27, 279–306 10.1146/annurev.neuro.27.070203.144130 15217334

[B74] Squire L. R. Wixted J. T. (2011). The cognitive neuroscience of human memory since H.M. Annu. Rev. Neurosci. 34, 259–288 10.1146/annurev-neuro-061010-113720 21456960 PMC3192650

[B75] Talland G. A. (1961). Confabulation in the Wernicke-Korsakoff syndrome. J. Nerv. Ment. Dis. 132, 361–381 10.1097/00005053-196105000-00001 13775041

[B76] Thorpe S. J. Rolls E. T. Maddison S. (1983). The orbitofrontal cortex: neuronal activity in the behaving monkey. Exp. Brain Res. 49, 93–115 10.1007/BF00235545 6861938

[B77] Treyer V. Buck A. Schnider A. (2003). Subcortical loop activation during selection of currently relevant memories. J. Cogn. Neurosci. 15, 610–618 10.1162/089892903321662985 12803971

[B78] Treyer V. Buck A. Schnider A. (2006). Selection of currently relevant words: an auditory verbal memory study using positron emission tomography. Neuroreport 17, 323–327 10.1097/01.wnr.0000199457.78670.44 16462606

[B79] Van der Horst L. (1932). Über die Psychologie des Korsakowsyndroms. Monatsschr. Psychiatr. Neurol. 83, 65–84 10.1159/000164016

[B80] Wahlen A. Nahum L. Gabriel D. Schnider A. (2011). Fake or fantasy: rapid dissociation between strategic content monitoring and reality filtering in human memory. Cereb. Cortex 21, 2589–2598 10.1093/cercor/bhr049 21459836

[B81] Wallis J. D. (2007). Orbitofrontal cortex and its contribution to decision-making. Annu. Rev. Neurosci. 30, 31–56 10.1146/annurev.neuro.30.051606.094334 17417936

